# Novel indazole-based small compounds enhance TRAIL-induced apoptosis by inhibiting the MKK7-TIPRL interaction in hepatocellular carcinoma

**DOI:** 10.18632/oncotarget.22614

**Published:** 2017-11-03

**Authors:** Ji-Yong Yoon, Jeong-Ju Lee, Sujin Gu, Myoung Eun Jung, Hyun-Soo Cho, Jung Hwa Lim, Soo Young Jun, Jun-Ho Ahn, Ju-Sik Min, Min-Hyuk Choi, Su-Jin Jeon, Yong-Jae Lee, Areum Go, Yun-Jeong Heo, Cho-Rok Jung, Gildon Choi, Kwangho Lee, Moon-Kook Jeon, Nam-Soon Kim

**Affiliations:** ^1^ Genome Research Center, Korea Research Institute of Bioscience and Biotechnology, Daejeon, 305-333, Republic of Korea; ^2^ Bio and Drug Discovery Division, Korea Research Institute of Chemical Technology, Daejeon, 34114, Republic of Korea; ^3^ Gene Therapy Research Unit, Korea Research Institute of Bioscience and Biotechnology, Daejeon, 305-333, Republic of Korea; ^4^ Department of Functional Genomics, Korea University of Science and Technology, Daejeon, 34113, Republic of Korea; ^5^ Medicinal Chemistry and Pharmacology, Korea University of Science and Technology, Daejeon, 34113, Republic of Korea

**Keywords:** TIPRL, apoptosis, HCC, TRAIL sensitizer

## Abstract

Hepatocellular carcinoma (HCC) is one of the most malignant tumors. Although various treatments, such as surgery and chemotherapy, have been developed, a novel alternative therapeutic approach for HCC therapy is urgently needed. Tumor necrosis factor-related apoptosis inducing ligand (TRAIL) is a promising anti-cancer agent, but many cancer cells are resistant to TRAIL-induced apoptosis. To help overcome TRAIL resistance in HCC cancer cells, we have identified novel chemical compounds that act as TRAIL sensitizers. We first identified the hit compound, TRT-0002, from a chemical library of 6,000 compounds using a previously developed high-throughput enzyme-linked immunosorbent assay (ELISA) screening system, which was based on the interaction of mitogen-activated protein kinase kinase 7 (MKK7) and TOR signaling pathway regulator-like (TIPRL) proteins and a cell viability assay. To increase the efficacy of this TRAIL sensitizer, we synthesized 280 analogs of TRT-0002 and finally identified two lead compounds (TRT-0029 and TRT-0173). Co-treating cultured Huh7 cells with either TRT-0029 or TRT-0173 and TRAIL resulted in TRAIL-induced apoptosis due to the inhibition of the MKK7-TIPRL interaction and subsequent phosphorylation of MKK7 and c-Jun N-terminal kinase (JNK). *In vivo*, injection of these compounds and TRAIL into HCC xenograft tumors resulted in tumor regression. Taken together, our results suggest that the identified lead compounds serve as TRAIL sensitizers and represent a novel strategy to overcome TRAIL resistance in HCC.

## INTRODUCTION

Hepatocellular carcinoma (HCC) is a severe human malignant tumor [1]. Although a surgery is believed to be an essential treatment for HCC, liver function eventually declines in most patients, resulting in poor survival rates. Many emerging therapeutic methods are available to improve the survival rate of patients with HCC, such as curative therapy, palliative therapy and various systemic treatments. However, these strategies have not dramatically improved outcomes for patients with HCC, and many researchers and drug companies consequently endeavor to develop alternative therapeutic approaches for the treatment of HCC [2, 3].

Tumor necrosis factor-related apoptosis-inducing ligand (TRAIL), a member of the tumor necrosis factor (TNF) superfamily, exhibits anti-cancer activity and selectively induces apoptosis in various cancer cells, but not in normal cells [4, 5]. TRAIL primarily binds to four receptors (i.e., death receptor (DR) 4/5 and decoy receptor (DcR) 1/2). DR4/5 contain a functional death domain (DD) to induce apoptosis signals, unlike DcR1/2. The binding of TRAIL to DR4/5 initiates an apoptosis signal by recruiting FAS-associated death domain (FADD) and DD, which consequently activates poly ADP ribose polymerase (PARP) and caspases [5–7]. However, many cancer cells are resistant to TRAIL-induced apoptosis, and these cells overexpress anti-apoptotic proteins (Bcl-2 and Bcl-XL), antagonistic receptors (DcR1/2), and defective FADD. The overexpression of cellular FLICE-inhibitory protein (cFLIP) and the loss of Bax and Bak are also related to TRAIL resistance in several types of cancer [8–10]. Various natural compounds that induce TRAIL-mediated apoptosis, such as chrysin, curcumin, and wogonin, have been reported to overcome TRAIL resistance in cancer cells [11–13]. However, the mode of action (MOA) by which many of these natural/small compounds increase TRAIL-induced apoptosis and act as TRAIL sensitizers is not known.

Our group recently reported that TOR signaling pathway regulator like (TIPRL) protein is overexpressed in HCC and contributes to TRAIL resistance by forming the mitogen-activated protein kinase kinase 7 (MKK7)-protein phosphatase type 2A (PP2Ac)-TIPRL complex. Furthermore, the knockdown of TIPRL by siRNA treatment activated the phosphorylation of MKK7 and c-Jun N-terminal kinase (JNK), which significantly promoted TRAIL-induced apoptosis in TRAIL resistant cell lines [8]. As a follow-up to this study, we established an *in vitro* enzyme-linked immunosorbent assay (ELISA) screening system based on the mechanism of the MKK7-TIPRL interaction to overcome TRAIL resistance. Using this system, we identified that *Tussilago farfara* L. (commonly known as coltsfoot) and *Taraxacum officinale* F.H. Wigg (commonly known as dandelion) are able to inhibit the MKK7-TIPRL interaction and act as TRAIL sensitizers [14, 15].

In this study, we screened more than 6,000 compounds to find inhibitors of the MKK7-TIPRL interaction using a high-throughput ELISA system, and discovered the target compound that is TRT-0002. Subsequently, we synthesized a series of 280 analogs and evaluated their TRAIL-sensitizing activity using cell-based cytotoxicity assays. After intensive hit-to-lead optimization, two lead compounds, TRT-0029 and TRT-0173, were found to enhance TRAIL-induced apoptosis in Huh7 cells by inhibiting MKK7-TIPRL interactions and activating MKK7/JNK. The TRAIL-sensitizing activity of the two lead compounds was also validated in an *in vivo* xenograft animal model. These results strongly suggest that the pharmacological inhibition of MKK7-TIPRL interaction is responsible for the effect of TIPRL gene knockdown on TRAIL sensitization, which is a promising strategy to overcome TRAIL resistance in HCC.

## RESULTS

### Screening of chemical compounds that enhance TRAIL-induced apoptosis by inhibiting MKK7-TIPRL interaction

**Figure 1:**
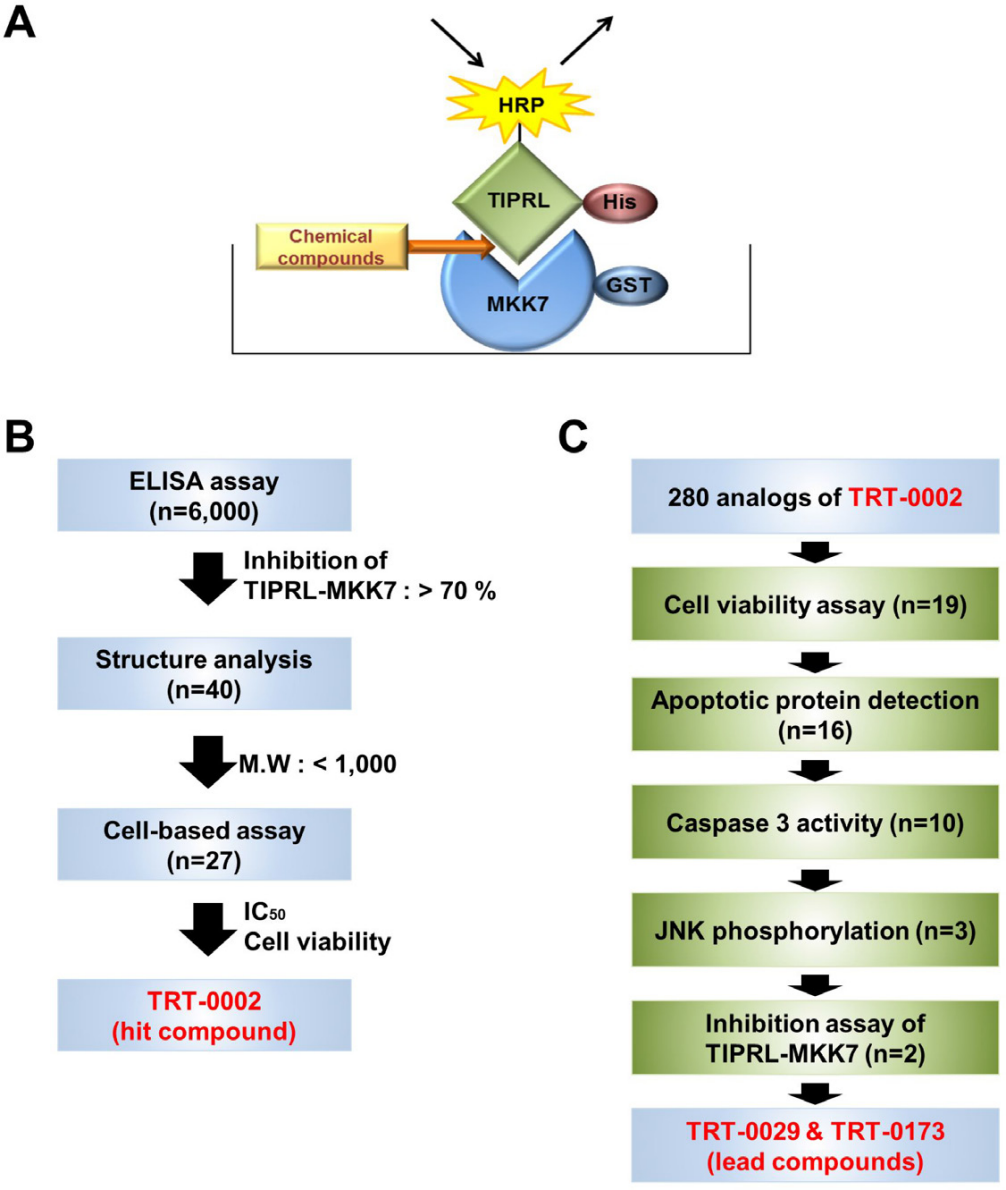
Strategies used to screen for chemical compounds that enhance TRAIL-induced apoptosis by inhibiting MKK7-TIPRL interaction (**A**) Schematic of an ELISA system designed to identify chemical compounds that inhibit the MKK7-TIPRL interaction. (**B**) Flowchart for the selection of the hit chemical compound, TRT-0002, from over 6,000 chemical compounds. (**C**) Flowchart for the selection of the lead compounds, TRT-0029 and TRT-0173, from 280 analogs of TRT-0002.

Using an ELISA system consisting of purified recombinant TIPRL and MKK7 proteins (Figure 1A) and the strategies shown in Figure 1B and 1C, we identified a novel chemical compound that inhibits the MKK7-TIPRL interaction. Over 6,000 compounds from an in-house chemical library were screened with the ELISA assay at 10 μM (Supplementary Figure 1A). Their average Z’value, a parameter used to measure the assay quality in high-throughput screens [16], exceeded 0.6, indicating that the assay was robust and reproducible (Supplementary Table 1). As initial hits in the ELISA assay, 40 compounds, in which the IC_50_ value was expected to be less than 10 μM, were selected according to their ability to inhibit the MKK7-TIPRL interaction by more than 70%. Then, these initial hits were re-evaluated in triplicate, and unwanted hits, such as natural products having high molecular weights (M.W. > 1,000), which compounds that could be difficult to optimize into orally bioactive drugs, or compounds that could have interfered with the ELISA assay, were removed. After that, dose-response curves were generated for the remaining 27 compounds, and their structures were analyzed to classify hit scaffolds. The potency (IC_50_) of these 27 compounds ranged from 0.03 μM to 20 μM (Supplementary Table 2). Subsequently, their TRAIL-sensitizing activities (i.e., their abilities to inhibit the growth of Huh7 cells in the presence of TRAIL) were tested. As shown in Supplementary Table 2, seven of the 27 compounds exhibited an IC_50_ of less than 1 mM, as demonstrated by *in vitro* ELISA. However, these seven compounds in the presence of TRAIL reduced the cell viability of Huh7 cells by no more than 20%. In contrast, four compounds (2, 16, 22, and 25) exhibited a synergetic effect in reducing the cell viability of Huh7 cells in the presence of TRAIL, indicating their potential as TRAIL sensitizers.

**Figure 2:**
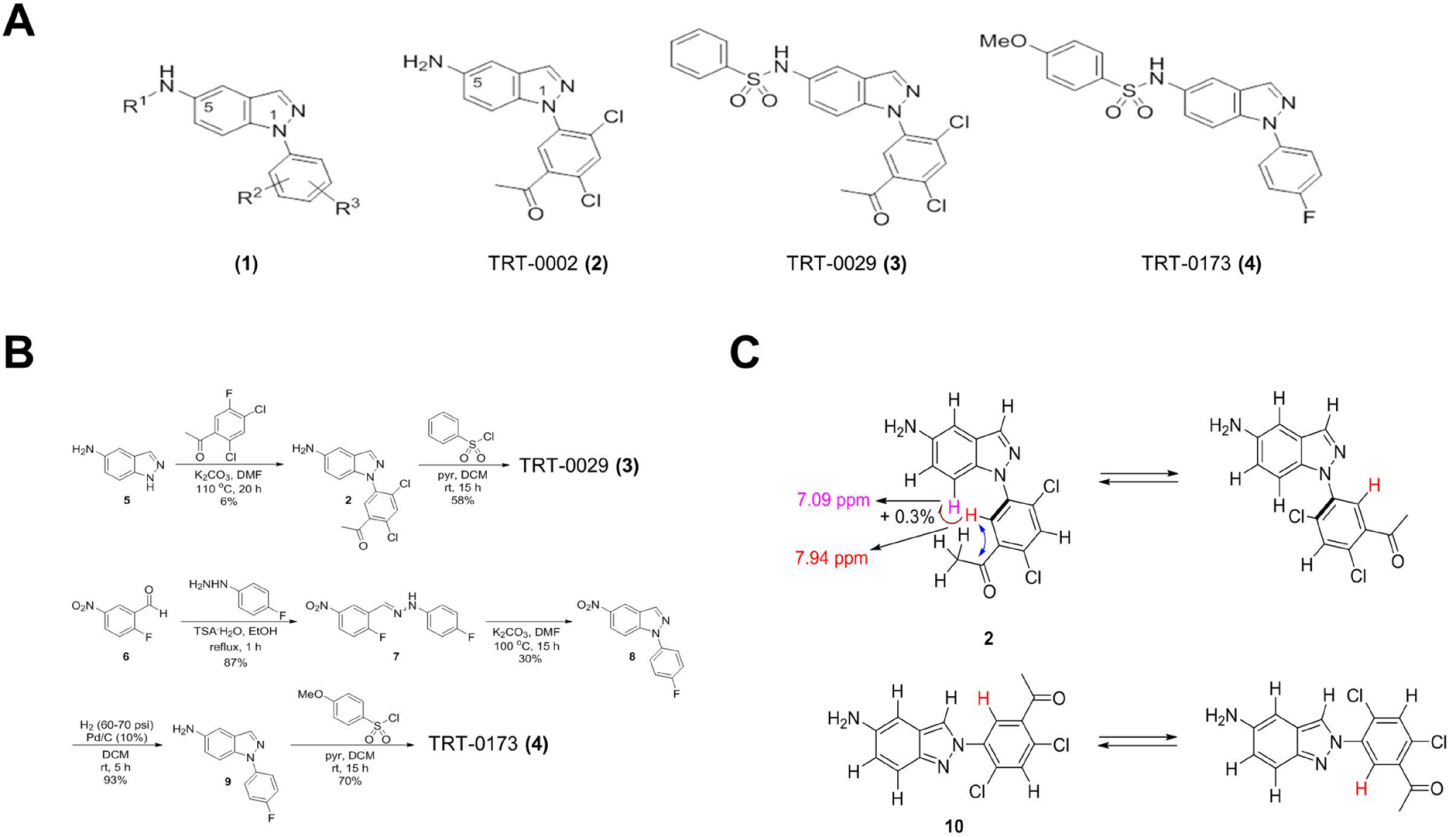
Synthesis and structure of chemical compounds (**A**) General structure (1) of indazole-based compounds and specific structures of TRT-0002 (2), TRT-0029 (3), and TRT-0173 (4). (**B**) The synthesis schemes for TRT-0029 (3) and TRT-0173 (4). (**C**) HMBC and NOE experimental results used to discriminate TRT-0002 (2) from its regioisomeric structure 10.

Importantly, among the four compounds, the compound 16 possesses the chemical structure with the most favorable drug-like properties. Thus, the compound 16 was selected as a hit compound; it was newly synthesized and named TRT-0002. The TRT-0002 compound, shown as (2) in Figure 2A, is a 1-aryl-5-aminoindozole, represented by the general structure (1). As shown in Supplementary Figure 1, TRT-0002 exhibited an IC_50_ of 1.4 μM to inhibit the MKK7-TIPRL interaction, and significantly enhanced TRAIL-induced cell death of Huh7 cells, compared to that with TRAIL alone (Supplementary Figure 1B and 1C). Western blot analysis further supports the premise that co-treatment of TRT-0002 and TRAIL reduces cell viability of Huh7 cells via the induction of apoptosis and JNK activation, as shown by the cleavages of caspase-3 and of PARP, as well as the phosphorylation of JNK (Supplementary Figure 1D and 1E).

To improve the potency of TRT-0002 as a TRAIL sensitizer, we synthesized a set of 280 analogs of TRT-0002, in which the initial medicinal chemistry effort was focused on variations of the aryl part at position 1 and the amino part at position 5 of its 1*H*-indazole core. Subsequently, 19 candidate chemical compounds were selected using an MTT assay of cell viability, in which the cell cytotoxicity by single compound was less than 60%, and the cell death by co-treatment of the compound and TRAIL, compared to compound only treatment, was more than 14% (Supplementary Table 3). Finally, we selected two lead compounds, TRT-0029 and TRT-0173, based on Western blot (Caspase-3, PARP, and JNK phosphorylation), caspase 3 activity assay, and glutathione S-transferase (GST) pull-down assay of MKK7-TIPRL interaction (Figure 1C and Supplementary Table 3); TRT-0029 and TRT-0173 were synthesized as shown in Figure 2B, and their ability to act as TRAIL sensitizers was tested.

### Lead compounds (TRT-0029 and TRT-0173) enhance TRAIL-induced Huh7 cell death

**Figure 3:**
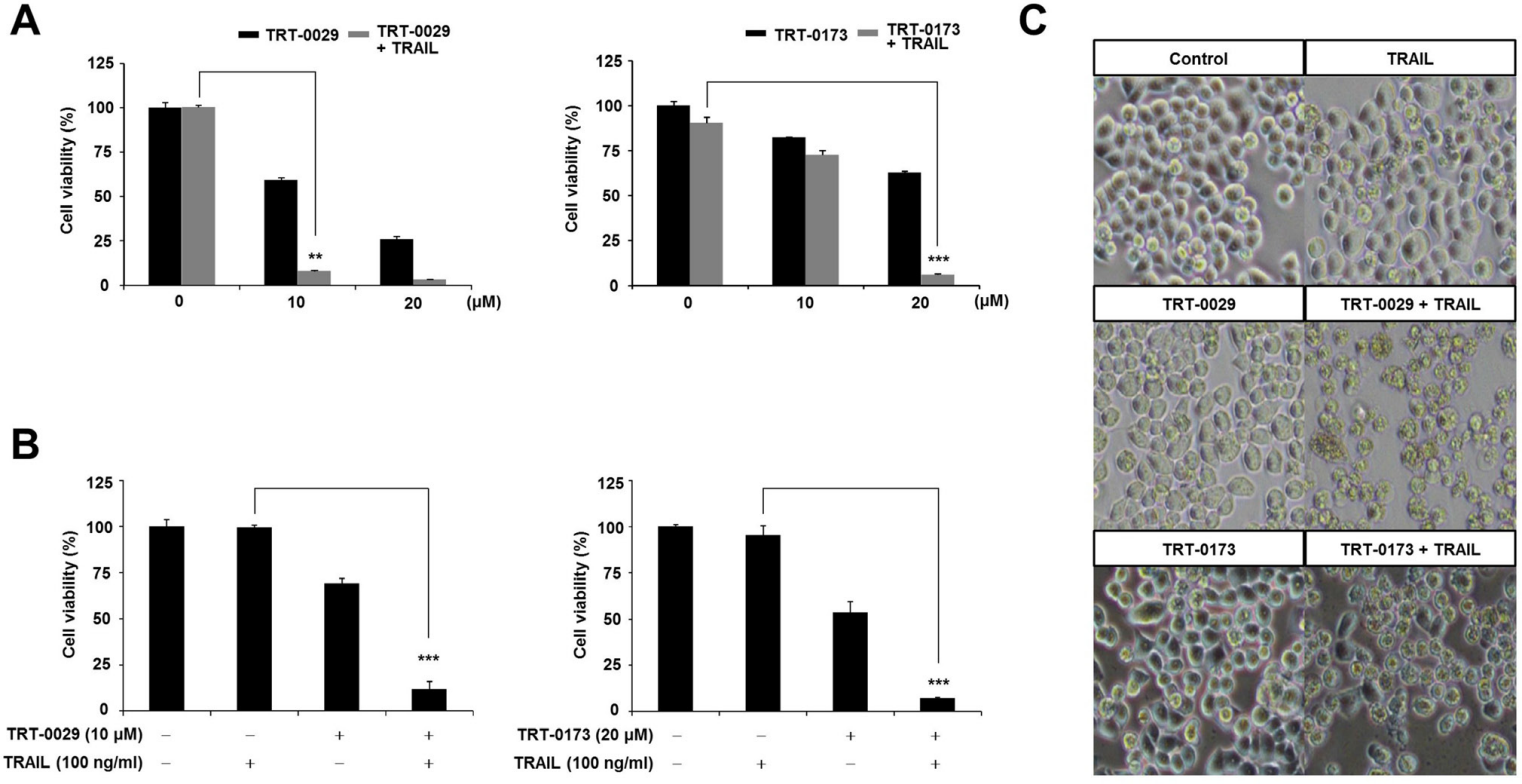
Lead compounds (TRT-0029 or TRT-0173) sensitize Huh7 cells to TRAIL-induced cell death (**A**) Huh7 cells were treated with various concentrations of lead compounds (TRT-0029 or TRT-0173) in the absence or presence of TRAIL (100 ng/ml) for 24 h, and an MTT assay was then performed. Differences in the viabilities of treated cells were assessed using Student's *t*-test (^**^p < 0.01, ^***^p < 0.001) (**B**) MTT assay for the viability of Huh7 cells treated with TRAIL alone, lead compound alone, and a combination of TRAIL and lead compound for 24 h. (^***^p < 0.001). (**C**) Morphologies characteristic of cell death were observed with a microscope. Pictures were taken at a magnification of ×100.

To assess the ability of the selected lead compounds to act as TRAIL sensitizers, we assessed cell viability with an MTT assay. To determine the effective working concentration of these compounds, Huh7 cells were first treated with various doses in the presence and absence of TRAIL; the effective working concentrations were determined to be 10 μM for TRT-0029 and 20 μM for TRT-0173 (Figure 3A). Consistently, when compared to that of TRAIL treatment alone, the co-treatment of these lead compounds (10 μM for TRT-0029 and 20 μM for TRT-0173) in the presence of TRAIL significantly reduced cell viability of Huh7 cells by nearly 90%. In addition, these co-treatments further enhanced the cell death of Huh7 cells by approximately 50% when compared to that with TRT-0002 and TRAIL co-treatment (Supplementary Figure 2A). We also confirmed that these compounds promoted cell morphologies characteristic of cell death in the presence of TRAIL, unlike cells that had been treated with the compounds alone (Figure 3C). These results indicate that TRT-0029 and TRT-0173 may act as TRAIL sensitizers.

To support our notion, we examined the cell viability of the HCC cell line, HepG2, and found that co-treatment with these lead compounds and TRAIL significantly decreased HepG2 viability by 36% for TRT-0029 and 53% for TRT-0173. However, this co-treatment did not induce cytotoxicity in WI-38 cells that were derived from normal human lung (Supplmentary Figure 3). Thus, these results support the HCC-specific ability of these lead compounds.

### Lead compounds enhance TRAIL-induced apoptosis via caspase signaling

**Figure 4:**
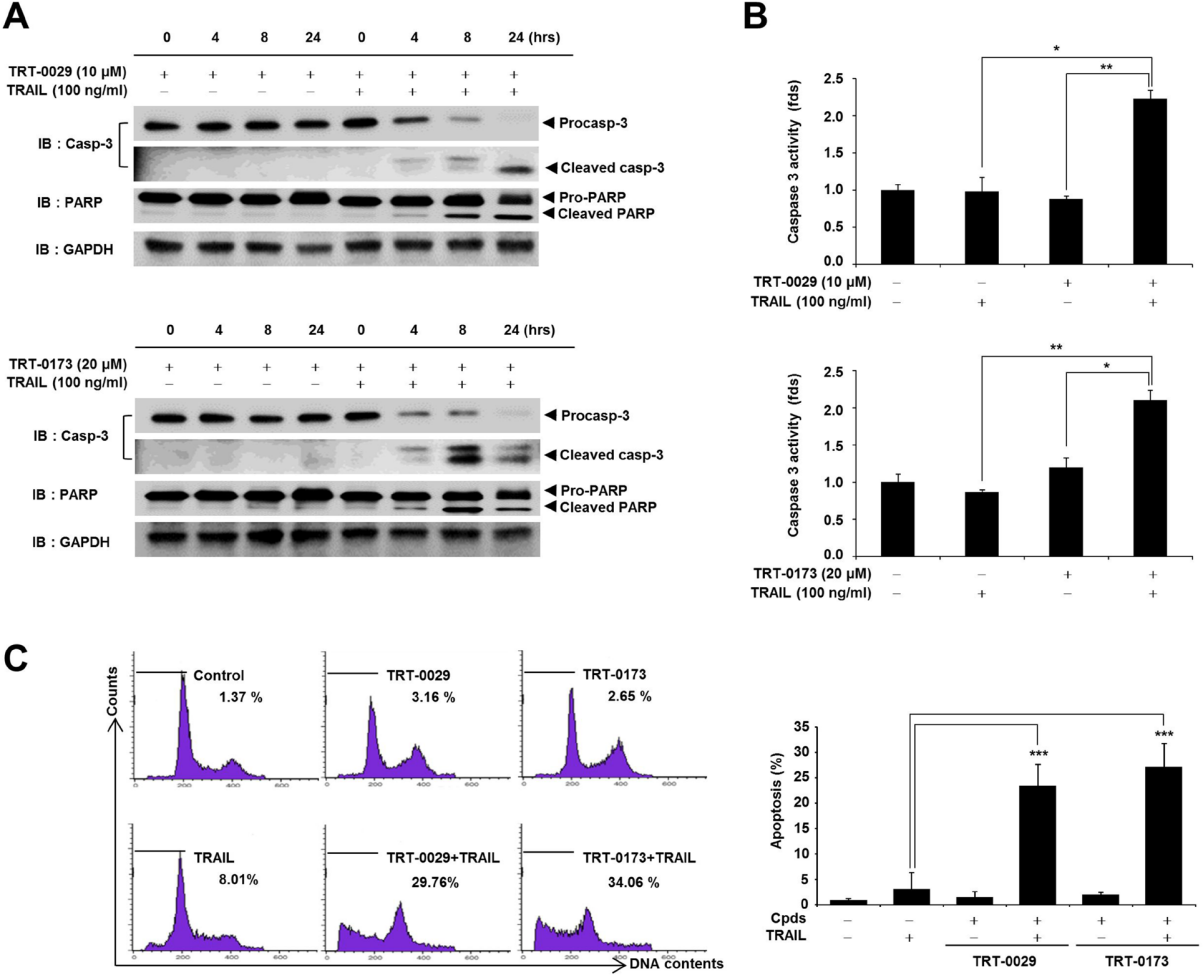
Lead compounds induce TRAIL-mediated apoptosis via caspase signals in Huh7 cells (**A**) Huh7 cells were treated with lead compound (10 μM for TRT-0029 and 20 μM for TRT-0173) in the presence or absence of 100 ng/ml TRAIL for the indicated time periods. The PARP and caspase-3 levels were examined by Western blotting. GAPDH was used as a loading control. (**B**) Huh7 cells were treated with lead compound (10 μM for TRT-0029 and 20 μM for TRT-0173) and TRAIL for 24 h. The cells were analyzed for caspase-3 activity and differences in this activity were assessed using Student's *t*-test (^*^p < 0.05, ^**^p < 0.01) (**C**) Cell cycle analysis of propidium iodide-stained Huh7 cells was performed using flow cytometry (left). The bars indicate the percentages of cells in the sub-G1 phase based on apoptosis signals. Bar graphs represent the percentages of sub-G1 DNA contents undergoing apoptosis (^***^p < 0.001) (right).

To identify the role of apoptosis in the cell death observed in response to co-treatment with the lead compounds and TRAIL, we investigated apoptosis markers, such as caspase-3 and PARP, using Western blotting. Specifically, co-treatment with TRT-0029 and TRAIL for 24 h effectively activated apoptosis signals (cleaved caspase-3 at 24 h and PARP at 8 h) compared with that of TRT-0029 alone (Figure 4A upper); TRAIL alone also did not activate caspase-3 (Supplementary Figure 2B). Additionally, the activity of caspase-3 was significantly increased in cells co-treated with TRT-0029 and TRAIL compared to that with TRT-0029 alone or TRAIL alone (Figure 4B upper). Combination treatment with TRT-0173 and TRAIL also activated apoptosis signals, similarly to the activation observed in response to TRT-0029 and TRAIL (Figure 4A and 4B lower). Furthermore, to validate that co-treatment with one of the lead compounds and TRAIL induced apoptosis, we performed a fluorescence-activated cell sorting (FACS) analysis with propidium iodide (PI) staining. Specifically, this treatment combination significantly increased the sub-G1 population at 24 h compared with that of cells treated with TRAIL alone (Figure 4C). These results indicate that our lead compounds enhance TRAIL-induced cell death by inducing apoptotic signaling.

### Lead compounds cause TRAIL-induced apoptosis by activating MKK7/JNK via the inhibition of the MKK7-TIPRL interaction

**Figure 5:**
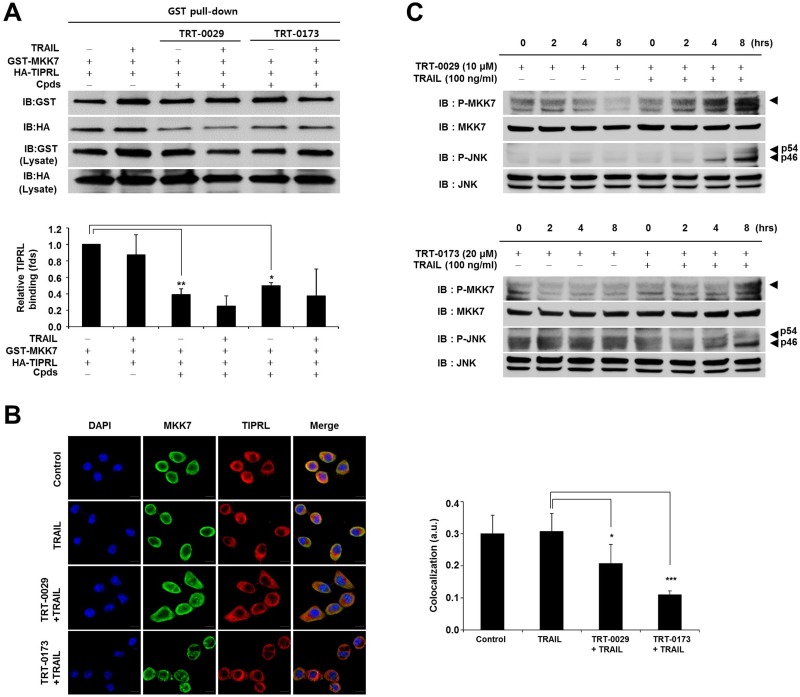
Effects of inhibition of MKK7-TIPRL interaction and activation of MKK7/JNK by the lead compounds (**A**) GST pull-down assay. 293T cells were co-transfected with GST-MKK7 and HA-TIPRL vectors for 24 h and then treated with lead compound (10 μM for TRT-0029 and 20 μM for TRT-0173) and/or TRAIL for 24 h (upper). The signal intensity corresponding to HA-TIPRL/GST-MKK7 was quantified by the ImageJ program, and differences in this activity were assessed using Student's *t*-test (^*^p < 0.05, ^**^p < 0.01) (lower) (**B**) Huh7 cells were treated with lead compound (10 μM for TRT-0029 and 20 μM for TRT-0173) in the presence of TRAIL for 8 h, were stained with antibodies against MKK7 (green) and TIPRL (red), and then observed by confocal microscopy. DAPI (blue) was used for nuclear staining. Scale bar, 50 μm (left). The significance of the differences was assessed with a one-way ANOVA and Bonferroni's multiple comparison post hoc test (^*^p < 0.05, ^***^p < 0.001). Yellow indicates the co-localization of TIPRL (red) and MKK7 (green) (right). a.u., artificial unit. (**C**) Huh7 cells were treated with lead compound in the presence or absence of 100 ng/ml TRAIL for the indicated time periods. The phosphorylation of MKK7 and JNK was examined by Western blotting.

We investigated the ability of the lead compounds to disrupt the interaction between MKK7 and TIPRL using a GST pull-down assay and immunocytochemical analysis. Figure 5A shows that treatment with TRT-0029 or TRT-0173 significantly decreased the interaction between MKK7 and TIPRL compared with that of untreated cells. In addition, immunocytochemical showed that co-treatment with one of the lead compounds and TRAIL significantly suppressed the endogenous interaction between MKK7 and TIPRL, as exemplified by the loss of co-localization of TIPRL (red) and MKK7 (green) (Figure 5B). These results suggest that the lead compounds may bind to the interaction regions of MKK7 and TIPRL, subsequently interfering with their interaction. Our previous report suggested that TRAIL induces apoptosis in TRAIL-resistant cells by activating MKK7/JNK phosphorylation via the inhibition of the MKK7-TIPRL interaction. Concordantly, we investigated the ability of co-treatment with one of the lead compounds and TRAIL to activate MKK7/JNK and observed the induction of MKK7/JNK phosphorylation in Huh7 cells (Figure 5C). Taken together, these results indicate that the lead compounds may disrupt the MKK7 and TIPRL interaction to facilitate TRAIL-induced apoptosis via the activation of MKK7/JNK.

### Lead compounds suppress tumor growth by cooperating with TRAIL in a mouse xenograft model

**Figure 6:**
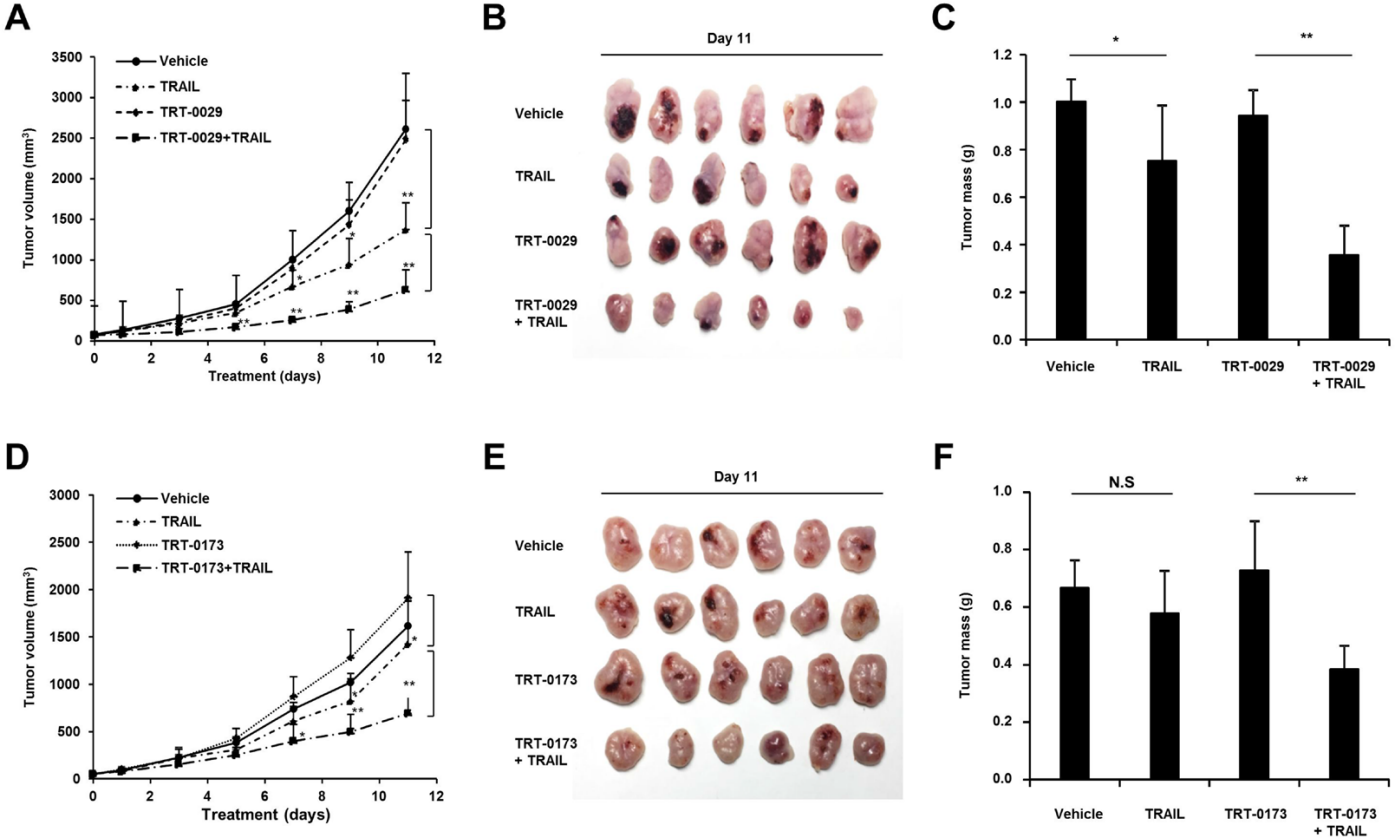

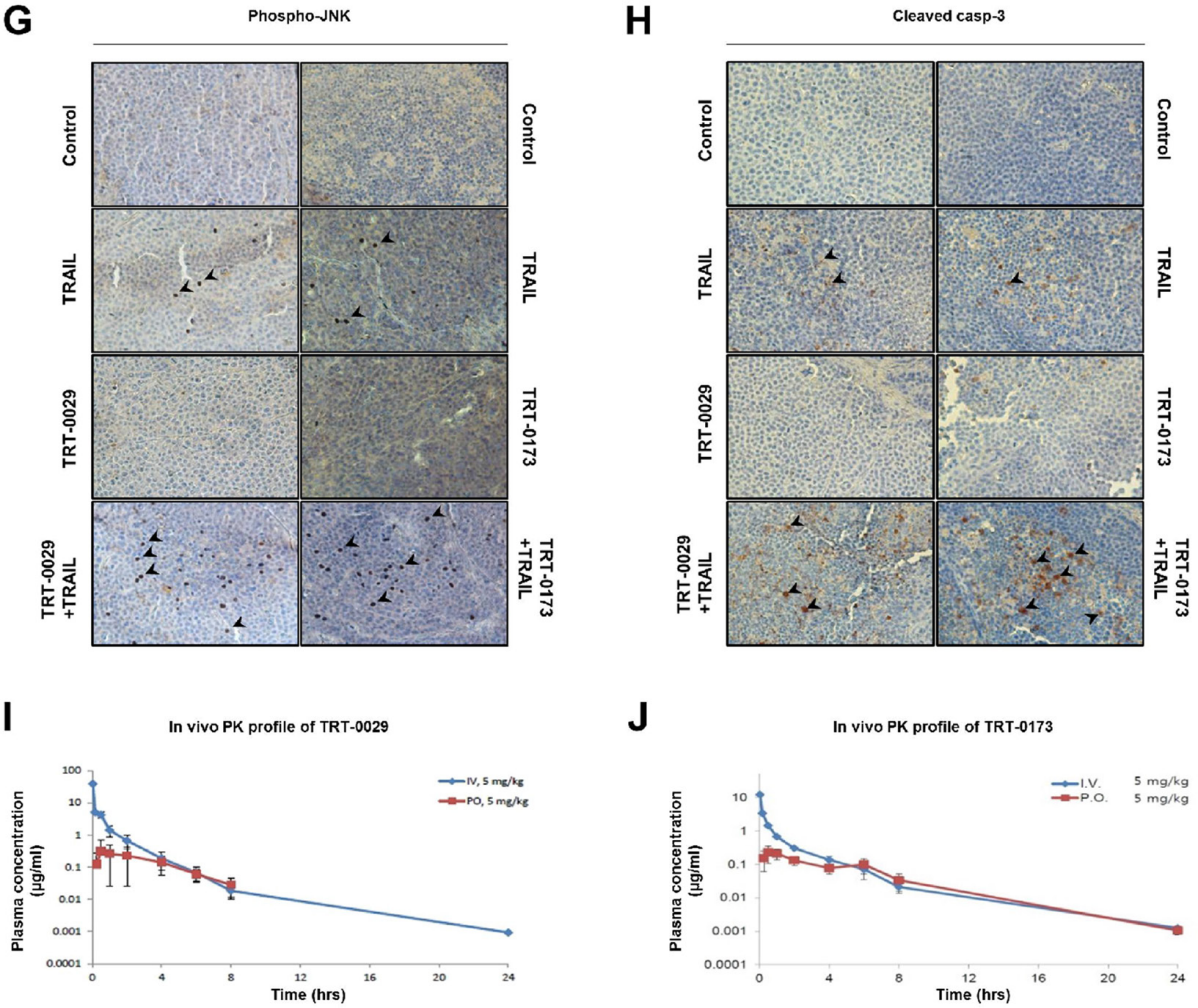
Treatment with lead compounds and TRAIL suppresses the growth of Huh7 tumor xenografts (**A**) and (**D**) Tumor formation was analyzed in an *in vivo* xenograft model in which Huh7 cells were subcutaneously injected. The nude mice were treated daily with vehicle, TRAIL (2.5 mg/kg, i.p), lead compound (30 mg/kg of TRT-0029: A or TRT-0173: D, i.v.), or lead compound in the presence of TRAIL for 11 days. Tumor volume was measured every other day for 11 days. Each point on the graph represents the average tumor volume ± SD (*n* = 6/group) (^*^p < 0.05, ^**^p < 0.01). (**B**) and (**E)** On the last day of study, the mice in the indicated groups were euthanized, and the tumors were excised and photographed. (**C**) and (**F**) Tumor mass was measured in each group at the time of euthanasia. Values are the means ± SD (^*^; *p* < 0.05, ^**^; *p* < 0.01, ns; non-significant). (**G**) and (**H**) Tumor sections from the tumors presented in B and E were stained with antibodies against p-JNK and cleaved caspase-3. The arrowheads indicate positive signals. (**I**) and (**J**) The drug plasma concentration was measured over time after i.v. tail vein (5 mg/kg) or oral (5 mg/kg) administration of lead compound (TRT-0029: I or TRT-0173: J).

We evaluated the anti-cancer activity of the lead compounds in combination with TRAIL in an *in vivo* Huh7 tumor model. At 11 days, tumor volume was significantly reduced in the combination treatment group of TRT-0029 and TRAIL compared with the tumor volume in the group treated with TRT-0029 alone (Figure 6A). After 11 days, the mice were euthanized, and the excised tumors are presented in Figure 6B. Interestingly, in contrast to the treatment with lead compounds or vehicle alone, which did not cause tumor shrinkage, the average tumor mass was reduced in the group treated with TRAIL alone (24.8 %), and this reduction was greater in the combination treatment group of TRT-0029 and TRAIL (64.5%) (Figure 6C). Moreover, the combination treatment of TRT-0173 and TRAIL moderately inhibited tumor growth compared with that of TRT-0173 alone by 11 days (Figure 6D and 6E). Similarly, tumor mass was reduced with combination treatment of TRT-0173 and TRAIL (42.2%) compared with that of vehicle control (Figure 6F). These results indicate that combination treatment with the lead compounds and TRAIL inhibited tumor growth more effectively than treatment with either lead compounds did *in vivo*. At 11 days after treatment, all groups of mice showed no significant differences in body weight (Supplementary Figure 4A and 4B), and there was no general cytotoxicity of the lead compounds, as indicated by hematoxylin and eosin (H&E) staining of liver tissue (Supplementary Figure 4C and 4D). In addition, immunohistochemistry of tumor tissues revealed increased JNK phosphorylation and apoptosis with combination treatment of the lead compounds and TRAIL, compared with that of lead compounds or TRAIL alone (Figure 6G and 6H). These findings suggest that the lead compounds suppress tumor growth by TRAIL-induced apoptosis though JNK activation.

Pharmacokinetic parameters of TRT-0029 and TRT-0173 were obtained in a rat model of intravenous (i.v., 5 mg/kg) and oral (p.o., 5 mg/kg) administration, as depicted in Figure 6I and 6J. The area under the curve (AUC) of TRT-0029 was 10.0 μg·h/ml (i.v.) and 1.22 μg·h/ml (p.o.) after 24 h. The oral availability of TRT-0029 was 11%. The AUC of TRT-0173 was 4.27 μg·h/ml (i.v.) and 1.15 μg·h/ml (p.o.) after 24 h, and its oral availability was 26.8%. Taken together, these *in vivo* results imply that the identified lead compounds (TRT-0029 and TRT-0173) exhibit HCC anti-tumor efficacy in cooperation with TRAIL based on their bioavailability in tumor tissues.

## DISCUSSION

TRAIL has been studied as a trigger of cell apoptosis in tumors with augmented growth and metastasis. Specifically, TRAIL targets DR4/5 receptors in the tumor to activate the caspase cascade. Because TRAIL is tumor specific, TRAIL treatment was associated with only mild side effects in clinical trials of non-small cell lung carcinoma and non-Hodgkin's lymphoma [17]. Thus, TRAIL is a promising anti-cancer drug for the treatment of several cancer types [4, 18]. However, various factors, such as the overexpression of DcR1/2, are involved in anti-apoptosis signaling. Therefore, cancer cells can acquire “TRAIL resistance”. In addition, breast cancer stem cells (bCSC) are also resistant to TRAIL treatment, but suppressing c-FLIP sensitizes these cells to TRAIL by derepressing the activity of the pro-apoptotic pathway [19]. Therefore, the development of TRAIL sensitizers is important to overcome the TRAIL resistance encountered in cancer treatment. To promote TRAIL sensitivity, many researchers have attempted to identify synergistic TRAIL sensitizers using high-throughput systems (HTSs) [20, 21]. Various small molecules, such as tunicamycin, bortezomib, geldanamycin, valproic acid, and cycloheximide, have been identified as TRAIL sensitizers in several cancer types [22–26]. Furthermore, 6-hydroxyflavanone (6-HF), 6-propionoxyflavanone (6-PF) [27], and 2-(5-methylselenophen-2-yl)-6,7-methylenedioxyquinolin-4-one (CCT327) [28] reportedly increases DR5 and decreases c-FLIP and Bcl-2. However, most studies of TRAIL sensitizers have been focused on the regulation of apoptotic and anti-apoptotic proteins, and the precise molecular mechanism underlying the efficacy of TRAIL sensitizer treatment remains unclear. Therefore, the precise molecular mechanisms of TRAIL sensitizing to enhance TRAIL treatment should be revealed.

Recently, we reported that TIPRL contributes to TRAIL resistance by mediating MKK7-PP2Ac interaction, and TIPRL knockdown enhances TRAIL-induced apoptosis by inhibiting this MKK7-PP2Ac interaction [8]. Furthermore, we identified TRAIL sensitizers from natural products, such as from *T. farfara* L. [14] and *T. officinale* F.H. Wigg [15], using an ELISA system that quantifies the MKK7-TIPRL interaction. Despite the many advantages of natural products and their extracts as therapeutic drugs, various unknown and heavy molecular weight components have hindered the development of anti-cancer drugs. Therefore, we attempted to identify small chemical compounds that act as TRAIL sensitizers in this study, because they rapidly diffuse across cell membranes and easily penetrate the blood-brain barrier. Using ELISA and a cell-based assay system, we screened over 6,000 small molecules and ultimately selected TRT-0002 as a hit compound. However, TRT-0002 is only effective at high concentrations and requires long treatment periods. Therefore, we attempted to identify a TRAIL sensitizer via TRT-0002 derivatization and consequently synthesized 280 analogs of TRT-0002. From this library, we identified the target compounds, TRT-0029 and TRT-0173, using various analysis systems.

To further confirm the ability of these lead compounds to act as TRAIL sensitizers, HepG2 cell lines, as well as a cell line from normal human lung (WI-38) were co-treated with TRAIL and the lead compounds. Importantly, the co-treatment of one of these leads with TRAIL caused a significant suppression of cell viability in HepG2 cells (Supplementary Figure 3A). This co-treatment exhibited higher efficacy in inducing cell death of Huh7 cells than did wogonin or curcumin, two previously reported TRAIL sensitizers (Supplementary Figure 2A). However, this co-treatment was not toxic to WI-38 cells, indicating the HCC-specific ability of these lead compounds and the lack of toxicity toward normal cells (Supplementary Figure 3B). This notion was further supported by the observation that the body weights and liver tissues of the mice co-injected with these lead compounds and TRAIL were not different from those of the mice treated with vehicle or TRAIL alone (Supplementary Figure 4). Collectively, these findings demonstrate the potential of these lead compounds as TRAIL sensitizers.

GST pull-down and immunocytochemical analyses demonstrated that co-treatment with TRAIL and TRT-0029 or TRT-0173 inhibited the MKK7-TIPRL interaction. Specifically, these compounds inhibited this interaction to facilitate TRAIL-induced apoptosis via the phosphorylation of MKK7/JNK. However, we did not examine the specific binding site of the lead compounds on TIPRL or MKK7. TRT-0029 and TRT-0173 could bind to either TIPRL or MKK7 to inhibit their interaction. Therefore, additional *in vitro* pull-down assays are needed to delineate the roles of TRT-0029 and TRT-0173 in this interaction.

Combination treatment with TRAIL and the lead compounds (TRT-0029 or TRT-0173) enhanced TRAIL-induced apoptosis in Huh7 cells. Moreover, to validate the cell-based results, we studied a Huh7 xenograft model *in vivo*. Co-treatment with TRAIL and the lead compounds (TRT-0029 or TRT-0173) suppressed tumor growth, indicating that these compounds act as TRAIL sensitizers. TRAIL alone showed dose-dependent anti-cancer effects *in vivo* at both 5 and 2.5 mg/kg (data not shown). This suggests that the dose of TRAIL *in vivo* must be carefully titrated. The anti-cancer effects of these compounds *in vivo* imply that they could serve as scaffolds to synthesize more effective derivatives that induce TRAIL-dependent growth arrest.

In conclusion, we identified two compounds (TRT-0029 and TRT-0173) using an ELISA system and cell-based study, and we confirmed that co-treatment of these compounds with TRAIL induced TRAIL-mediated apoptosis in Huh7 cells. These results suggest that TRT-0029 and TRT-0173 can serve as new TRAIL sensitizers for TRAIL-based cancer therapy. Furthermore, our screening pipeline system could be applied to identify novel TRAIL sensitizers in small compound/ natural product libraries.

## MATERIALS AND METHODS

### ELISA assay

The full-length cDNAs of human TIPRL and MKK7 were provided by the Korea Human Gene Bank (Korea Research Institute of Biosciences and Biotechnology, South Korea). TIPRL and MKK7 were subcloned into the pET21a vector (purchased from Novagen, San Diego, CA) and the pEBG vector containing a GST tag (kindly provided by Y. Liu, National Institute on Aging, National Institutes of Health) to construct the His-tagged and the GST-tagged plasmids, respectively. Purified TIPRL was conjugated with horseradish peroxidase (HRP). To screen chemical compounds using HTS, an ELISA system that detected the TIPRL and MKK7 interaction was constructed. Briefly, 96-well ELISA plates were coated with GST-MKK7 dissolved in PBS-T buffer (PBS containing 0.05% Tween-20 and 0.5% BSA), followed by the addition of 100 μl of TIPRL-HRP dissolved in PBS-T buffer. After 1 h, 100 μl of tetramethylbenzidine (TMB) solution was added to each well and the absorbance was read at 450 nm.

### Synthesis of chemical compounds

TRT-0029 and TRT-0173 were synthesized as shown in Figure 2B and 2C. The arylation of 5-amino-1*H*-indazole (5) with 1-acetyl-2, 4-dichloro-5-fluorobenzene under basic conditions (K_2_CO_3_, DMF, 110°C) yielded the intermediate 2. Compound 2 was sulfonylated with benzenesulfonyl chloride under standard conditions to afford TRT-0029 (Figure 2A). The condensation reaction of 2-fluoro-5-nitrobenzaldehyde (6) with 4-fluorophenylhydrazine in the presence of toluenesulfonic acid resulted in the formation of the hydrazone 7, which was then cyclized in the presence of potassium carbonate to give the 5-nitro-1*H*-indazole 8. The nitro reduction of compound 8 and subsequent sulfonylation with 4-methoxybenzenesulfonyl chloride yielded TRT-0173 (Figure 2B lower). The structure of intermediate 2 was discriminated from its 2-aryl-2*H*-indazole regioisomer 10 based on heteronuclear multiple bond correlation (HMBC) and nuclear Overhauser effect (NOE) experiments, as shown in Figure 2C. Briefly, the structures were identified as follows. The singlet proton at 7.94 ppm was assigned at the position 6 of the 5-acetyl-2,4-dichlorophenyl moiety because it correlated with the acetyl carbonyl carbon at 198.6 ppm in the HMBC spectrum. The proton exhibited 0.3% NOE on a doublet proton at 7.09 ppm, which was assigned at position 7 of the indazole core structure. Structure 10, a regioisomer of 2, would exhibit different behavior in the NOE experiment.

### Reagents

Antibodies against phospho-MKK7 (p-MKK7, Ser271/Thr275), phospho-JNK (p-JNK, Thr183/ Tyr185), JNK, caspase-3, cleaved caspase-3, and PARP were purchased from Cell Signaling Technology (Beverly, MA, USA). Antibodies against GST, hemagglutinin, and GAPDH, as well as HRP-conjugated secondary antibodies were obtained from AbFrontier (Seoul, South Korea). Human TRAIL was a kind gift from Dr. Choi (KAIST, South Korea), and was also purchased from Apotech (Chemin Des Croisettes, Switzerland).

### Cell culture

Huh7, 293T, HepG2, and WI-38 cells (American Type Culture Collection, Manassas, USA) were maintained in Dulbecco's modified Eagle medium (DMEM) or minimum essential medium (MEM), supplemented with 10% fetal bovine serum (FBS) and 1% penicillin/streptomycin in a humidified atmosphere in a 5% CO_2_ incubator at 37°C.

### Cell viability assay

Cell viability was assessed using a 3-(4,5-dimethylthiazol-2-yl)-2,5-diphenyltetrazolium bromide (MTT, Sigma-Aldrich, St. Louis, USA) assay. The cells were seeded at a density of 1 × 10^4^ cells per well and treated with the lead compounds and/or TRAIL. After 24 or 48 h, MTT (2 mg/ml) was added to each well, and the absorbance was measured using a microplate reader (Bio-Rad, Hercules, USA) at 570 nm. The cell viability was calculated as the percentage of viable cells in the drug-treated group versus the untreated control using the following equation: cell viability (%) = [OD (drug)-OD (blank)]/[OD (control)-OD (blank)] × 100.

### Cell cycle analysis

The cells were fixed in 70% ethanol at −20 °C and treated with 10 mg/ml RNase A for 1 h at 37°C. Next, the pellets were suspended in 1 ml of PI solution (50 μg/ml PI, 1 mg/ml RNase A, and 0.1% (w/v) Triton™ X-100 in 3.8 mM sodium citrate). The samples were analyzed using Cell Quest Software (BD Bio-sciences, San Jose, USA).

### Caspase 3 activity assay

The caspase 3 activity was measured using a caspase-3 luminescent assay kit (Promega, Madison, WI, USA), according to the manufacturer's instructions, and a luminescence plate reader (Molecular Devices Co., Sunnyvale, USA).

### GST pull-down assay

The full-length cDNAs of human TIPRL and MKK7 were subcloned into the pCGN vector (W. Herr, Cold Spring Harbor Laboratory) and the pEBG vector containing a GST tag to construct the HA-tagged and the GST-tagged plasmids, respectively. 293T cells were transfected with expression vector constructs using the TurboFect Transfection Reagent (Thermo Fisher Scientific, Lafayette, USA) and then were lysed in RIPA buffer (50 mM Tris-HCl, 150 mM NaCl, 1% Triton™ X-100, 0.1% SDS, 1 mM EDTA, 1 mM Na_3_VO_4_, and 1 mM NaF) containing complete protease inhibitor cocktail (Roche Applied Science, Indianapolis, USA). Whole-cell extracts were incubated with Glutathione Sepharose 4B (GE Healthcare, Uppsala, Sweden), and the eluted GST-tagged proteins were subjected to Western blotting using the indicated antibodies.

### Western blot analysis

The cells were lysed in lysis buffer (50 mM Tris-HCl, pH 7.4, 150 mM NaCl, 1% Triton™ X-100, 0.1% SDS, 1 mM EDTA, 1 mM Na_3_VO_4_, 1 mM NaF, and protease inhibitor cocktail) and clarified by centrifugation at 14,000×g for 20 min at 4°C. The protein samples were then subjected to Western blotting with the indicated antibodies.

### Immunocytochemistry

Cells were fixed/permeabilized using Molecular Probes fixative/permeabilization solution (Life technologies, Carlsbad, CA, USA) and incubated with antibodies against MKK7 and TIPRL. For subsequent confocal observation (Carl Zeiss), the primary antibodies were detected with Alexa Fluor^®^ 488 goat anti-mouse (MKK7) and Alexa Fluor^®^ 568 goat anti-rabbit (TIPRL) antibodies. The co-localization of TIPRL/MKK7 was quantified using ZEN imaging software (Carl Zeiss) to calculate Mander's overlap coefficient.

### Mice

Five-week-old female BALB/c nude mice were purchased from SLC Japan and were maintained in accordance with the guidelines of the Institutional Review Committee for Animal Care and Use of the Korea Research Institute of Bioscience and Biotechnology. Protocol approval was obtained from the same committee.

### *In Vivo* xenograft Assay

Huh7 cells were harvested and subcutaneously injected (5 × 10^6^ cells/mouse) into nude mice (N = 6/group for TRT-0173 or TRT-0029 experiments). When the tumor volume reached ~100 mm^3^, the mice were divided into four groups and treated with vehicle, TRAIL (2.5 mg/kg, intraperitoneal), lead compound (TRT-0029 or TRT-0173, 30 mg/kg, intravenously), or lead compound (30 mg/kg, intravenously) combined with TRAIL (2.5 mg/kg, intraperitoneal) daily for 11 days (11 treatments in total). The tumor volumes were measured every 2 days for 11 days after treatment, and tumor volume was calculated according to the following equation: V (mm^3^) = width^2^ (mm^2^) × length (mm)/2. Body weight and tumor mass were measured 11 days after treatment.

### Immunohistochemistry

We used the DAKO EnVision System for immunohistochemistry and following solutions were obtained from DAKO (Carpinteria, CA, USA). Tumors excised from mice were fixed in 10% formalin solution, embedded in paraffin, and sectioned. After deparaffinization, antigens were retrieved by heating the sections in Target Retrieval Solution (pH 9.0). To eliminate endogenous peroxidase, sections were blocked with Peroxidase Blocking Solution for 10 min. After 30 min of protein blocking, sections were incubated with anti-p-JNK and anti-cleaved-caspase 3 antibodies overnight. After washing with PBS, the secondary antibody incubation was carried out using DAKO Labeled Polymer HRP anti-rabbit for 1 h. The reaction was visualized by treatment with 3,3’-diaminobenzidine (DAB) stain. After optimal color was achieved, the sections were immediately washed and dehydrated in increasing concentrations of ethanol, and finally in xylene. Sections were observed with a microscope after mounting.

### H&E staining

Liver tissues dissected from mice were fixed in 10% formalin solution, embedded in paraffin, and sectioned. Liver tissue sections were stained with H&E. Sections were observed with a microscope after mounting.

### Pharmacokinetics

Male Sprague-Dawley rats anesthetized with ketamine were cannulated with polyethylene tubing in the femoral vein. A solution of lead compound (TRT-0029 or TRT-0173) was prepared by dissolving the compounds in a mixture of dimethyl sulfoxide (DMSO)/ polyethylene glycol 400 (PEG 400)/ distilled water (0.5: 4: 5.5), and was administered to rats by a bolus injection in the femoral vein. Blood samples were collected via the femoral vein 0, 2, 10, and 30 min, as well as 1, 2, 4, 6, 8, and 24 h after intravenous administration. For oral administration, blood was collected 0.25, 0.5, 1, 2, 4, 6, 8, and 24 h after treatment. The plasma fractions were prepared by centrifugation and were stored at −70°C before the liquid chromatography-tandem mass spectrometry (LC/MS/MS) analysis. The concentrations of target compound in the plasma were measured using LC-MS/MS (4000 QTRAP, AB SCIEX, Foster City, CA) in multiple reaction monitoring (MRM) mode. The PK parameters were determined with non-compartmental methods using Phoenix WinNolin (Pharsight, Mountain View, CA). The area under the plasma concentration-time curve (AUC) was calculated using the trapezoidal rule extrapolated to infinity. The terminal elimination half-life (t_1/2_), and the systemic clearance (CL) were obtained. The peak plasma concentration (C_max_) after oral administration was obtained by visual inspection of the plasma concentration-time plot of each rat.

### Statistical analysis

All data are presented as the means ± standard deviation (S.D.) of three independent experiments. The significance of differences was verified with Student's *t*-test using Sigmaplot software (Systat Software Inc., San Jose, CA, USA).

## SUPPLEMENTARY MATERIALS FIGURES AND TABLES



## Abbreviations

TIPRL: TOR signaling pathway regulator-like protein
TRAIL: TNF-related apoptosis-inducing ligand
ELISA: Enzyme-linked immunosorbent assay
GST: Glutathione S-Transferase
HCC: hepatocellular carcinoma cells
JNK: c-Jun N-terminal kinase
MKK7: mitogen-activated protein kinase kinase 7
PARP: poly ADP ribose polymerase

## References

[1] Arzumanyan A, Reis HM, Feitelson MA (2013). Pathogenic mechanisms in HBV- and HCV-associated hepatocellular carcinoma. Nat Rev Cancer.

[2] Johnson PJ (2005). Non-surgical treatment of hepatocellular carcinoma. HPB (Oxford).

[3] Lopez PM, Villanueva A, Llovet JM (2006). Systematic review: evidence-based management of hepatocellular carcinoma--an updated analysis of randomized controlled trials. Aliment Pharmacol Ther.

[4] Holoch PA, Griffith TS (2009). TNF-related apoptosis-inducing ligand (TRAIL): a new path to anti-cancer therapies. Eur J Pharmacol.

[5] Wang S, El-Deiry WS (2003). TRAIL and apoptosis induction by TNF-family death receptors. Oncogene.

[6] Elmallah MI, Micheau O (2015). Marine Drugs Regulating Apoptosis Induced by Tumor Necrosis Factor-Related Apoptosis-Inducing Ligand TRAIL. Mar Drugs.

[7] Wajant H, Pfizenmaier K, Scheurich P (2002). TNF-related apoptosis inducing ligand (TRAIL) and its receptors in tumor surveillance and cancer therapy. Apoptosis.

[8] Song IS, Jun SY, Na HJ, Kim HT, Jung SY, Ha GH, Park YH, Long LZ, Yu DY, Kim JM, Kim JH, Ko JH, Kim CH (2012). Inhibition of MKK7-JNK by the TOR signaling pathway regulator-like protein contributes to resistance of HCC cells to TRAIL-induced apoptosis. Gastroenterology.

[9] Zhang L, Fang B (2005). Mechanisms of resistance to TRAIL-induced apoptosis in cancer. Cancer Gene Ther.

[10] Zhu J, Zhou Q, Tan S (2016). Targeting miRNAs associated with surface expression of death receptors to modulate TRAIL resistance in breast cancer. Cancer Lett.

[11] Deeb D, Jiang H, Gao X, Hafner MS, Wong H, Divine G, Chapman RA, Dulchavsky SA, Gautam SC (2004). Curcumin sensitizes prostate cancer cells to tumor necrosis factor-related apoptosis-inducing ligand/Apo2L by inhibiting nuclear factor-kappaB through suppression of IkappaBalpha phosphorylation. Mol Cancer Ther.

[12] Ding J, Polier G, Kohler R, Giaisi M, Krammer PH, Li-Weber M (2012). Wogonin and related natural flavones overcome tumor necrosis factor-related apoptosis-inducing ligand (TRAIL) protein resistance of tumors by down-regulation of c-FLIP protein and up-regulation of TRAIL receptor 2 expression. J Biol Chem.

[13] Lirdprapamongkol K, Sakurai H, Abdelhamed S, Yokoyama S, Athikomkulchai S, Viriyaroj A, Awale S, Ruchirawat S, Svasti J, Saiki I (2013). Chrysin overcomes TRAIL resistance of cancer cells through Mcl-1 downregulation by inhibiting STAT3 phosphorylation. Int J Oncol.

[14] Lee HJ, Cho HS, Jun SY, Lee JJ, Yoon JY, Lee JH, Song HH, Choi SH, Kim SY, Saloura V, Park CG, Kim NS (2014). Tussilago farfara L. augments TRAIL-induced apoptosis through MKK7/JNK activation by inhibition of MKK7TIPRL in human hepatocellular carcinoma cells. Oncol Rep.

[15] Yoon JY, Cho HS, Lee JJ, Lee HJ, Jun SY, Lee JH, Song HH, Choi S, Saloura V, Park CG, Kim CH, Kim NS (2016). Novel TRAIL sensitizer Taraxacum officinale F.H. Wigg enhances TRAIL-induced apoptosis in Huh7 cells. Mol Carcinog.

[16] Zhang JH, Chung TD, Oldenburg KR (1999). A Simple Statistical Parameter for Use in Evaluation and Validation of High Throughput Screening Assays. J Biomol Screen.

[17] Kruyt FA (2008). TRAIL and cancer therapy. Cancer Lett.

[18] Oikonomou E, Pintzas A (2013). The TRAIL of oncogenes to apoptosis. Biofactors.

[19] Piggott L, Omidvar N, Marti Perez S, French R, Eberl M, Clarkson RW (2011). Suppression of apoptosis inhibitor c-FLIP selectively eliminates breast cancer stem cell activity in response to the anti-cancer agent TRAIL. Breast Cancer Res.

[20] Booth NL, Sayers TJ, Brooks AD, Thomas CL, Jacobsen K, Goncharova EI, McMahon JB, Henrich CJ (2009). A cell-based high-throughput screen to identify synergistic TRAIL sensitizers. Cancer Immunol Immunother.

[21] Finlay D, Richardson RD, Landberg LK, Howes AL, Vuori K (2010). Novel HTS strategy identifies TRAIL-sensitizing compounds acting specifically through the caspase-8 apoptotic axis. PLoS One.

[22] Jiang CC, Chen LH, Gillespie S, Kiejda KA, Mhaidat N, Wang YF, Thorne R, Zhang XD, Hersey P (2007). Tunicamycin sensitizes human melanoma cells to tumor necrosis factor-related apoptosis-inducing ligand-induced apoptosis by up-regulation of TRAIL-R2 via the unfolded protein response. Cancer Res.

[23] Seol DW (2011). p53-Independent up-regulation of a TRAIL receptor DR5 by proteasome inhibitors: a mechanism for proteasome inhibitor-enhanced TRAIL-induced apoptosis. Biochem Biophys Res Commun.

[24] Ma Y, Lakshmikanthan V, Lewis RW, Kumar MV (2006). Sensitization of TRAIL-resistant cells by inhibition of heat shock protein 90 with low-dose geldanamycin. Mol Cancer Ther.

[25] Kim HB, Kim MJ, Lee SH, Lee JW, Bae JH, Kim DW, Dao TT, Oh WK, Kang CD, Kim SH (2012). Amurensin G, a novel SIRT1 inhibitor, sensitizes TRAIL-resistant human leukemic K562 cells to TRAIL-induced apoptosis. Biochem Pharmacol.

[26] Sah NK, Munshi A, Kurland JF, McDonnell TJ, Su B, Meyn RE (2003). Translation inhibitors sensitize prostate cancer cells to apoptosis induced by tumor necrosis factor-related apoptosis-inducing ligand (TRAIL) by activating c-Jun N-terminal kinase. J Biol Chem.

[27] Szliszka E, Kostrzewa-Suslow E, Bronikowska J, Jaworska D, Janeczko T, Czuba ZP, Krol W (2012). Synthetic flavanones augment the anticancer effect of tumor necrosis factor-related apoptosis-inducing ligand (TRAIL). Molecules.

[28] Liu YJ, Lin YC, Lee JC, Kuo SC, Ho CT, Huang LJ, Kuo DH, Way TD (2014). CCT327 enhances TRAIL-induced apoptosis through the induction of death receptors and downregulation of cell survival proteins in TRAIL-resistant human leukemia cells. Oncol Rep.

