# Supplementary material for: Novel indazole-based small compounds enhance TRAIL-induced apoptosis by inhibiting the MKK7-TIPRL interaction in hepatocellular carcinoma
Source: Oncotarget. 2017 Nov 3;8(68):112610–22. doi: 10.18632/oncotarget.22614 (PMC5762535; doi:10.18632/oncotarget.22614)
Supplement: Supplementary file 1 [file oncotarget-08-112610-s001.pdf]

Novel indazole-based small compounds enhance TRAIL-induced apoptosis by inhibiting the MKK7-TIPRL interaction in hepatocellular carcinoma

SUPPLEMENTARY MATERIALS

MATERIALS AND METHODS

Preparation of 1-(5-acetyl-2,4-dichlorophenyl)-5-amino-1H-indazole (1)

A mixture of 5-amino-1*H*-indazole (6.40 g, 48.1 mmol), 1-acetyl-2,4-dichloro-5-fluorobenene (10.0 g, 48.3 mmol), and potassium carbonate (66.8 g, 483 mmol) in *N,N*-dimethylformamide (200 mL) was heated to 110 °C for 20 h. After cooling to room temperature, the reaction mixture was partitioned between dichloromethane and water, and the organic layer was washed several times with water and dried over magnesium sulfate. The solvent was evaporated in vacuo and the residue was separated by chromatography on a silica gel column (*n*-hexane : dichloromethane : ethyl acetate = 1 : 5 : 1) to give the intermediate **1** (1.00 g, 6 %). ^{1}H NMR (500 MHz, $CDCl_3$): δ 8.11 (s, 1 H), 8.05 (s, 1 H), 7.94 (s, 1 H), 7.09 (d, *J* = 8.5 Hz, 1 H), 6.88 (d, *J* = 8.5 Hz, 1 H), 6.86 (s, 1 H), 5.02 (br s, 2 H), 2.63 (s, 3 H); MS (ESI): *m/z* for $C_{15}H_{11}Cl_2N_3O$, found 320 [M+H$^+$].

Preparation of 1-(5-acetyl-2,4-dichlorophenyl)-5-benzenesulfonamido-1H-indazole (3, TRT-0029)

Benzenesulfonyl chloride (14 *µL*, 0.11 mmol) was added to a solution of compound 1 (30 mg, 0.094 mmol) and pyridine (11 µL, 0.14 mmol) in dichloromethane (2 mL) at room temperature and the resulting mixture was stirred at the same temperature for 15 h. The reaction mixture was partitioned between dichloromethane and water, and the organic layer was dried over magnesium sulfate. The solvent was evaporated in vacuo, and the residue was separated by chromatography on a silica gel column (*n*-hexane : ethyl acetate = 4 : 1) to give the final product 3 (25 mg, 58 %). ^{1}H NMR (500 MHz, $CDCl_3$): δ 8.18 (s, 1 H), 7.76 (t, *J* = 7.5 Hz, 3 H), 7.72 (s, 1 H), 7.57-7.54 (m, 2 H), 7.46 (t, *J* = 8.00 Hz, 2 H), 7.13 (d, *J* = 1.5 Hz, 2 H), 6.69 (s, 1 H), 2.70 (s, 3 H); MS (ESI): *m/z* for $C_{21}H_{15}Cl_2N_3O_3S$, found 460 [M+H$^+$].

Preparation of 1-(2-fluoro-5-nitrobenzylidene)-2-(4-fluorophenyl)hydrazine (7)

4-fluorophenylhydrazine (37 mg, 0.29 mmol) and toluenesulfonic acid monohydrate (3.0 mg, 0.02 mmol) were added to a solution of 2-fluoro-5-nitrobenzaldehyde (50 mg, 0.27 mmol) in ethanol, and the mixture was stirred at reflux for 1 h. After cooling to room temperature, the precipitated solid was collected by filtration to give the hydrazone **7** (65 mg, 87 %). ^{1}H NMR (300 MHz, $CDCl_3$): δ 8.84 (dd, *J* = 6.2, 2.9 Hz, 1 H), 8.12 (m, 1 H), 7.86 (s, 1 H), 7.23 (t, *J* = 9.3 Hz, 1 H), 7.08 (m, 4 H); MS (ESI): *m/z* for $C_{13}H_9F_2N_3O_2$, found 278 [M+H$^+$].

Preparation of 1-(4-fluorophenyl)-5-nitro-1H-indazole (8)

A mixture of compound **7** (61 mg, 0.22 mmol) and potassium carbonate (134 mg, 0.97 mmol) in *N,N*-dimethylformamide (2 mL) was heated to 100 °C for 15 h. After cooling to room temperature, the reaction mixture was partitioned between ethyl acetate and water, and the organic layer was washed with water several times and dried over magnesium sulfate. The solvent was evaporated in vacuo, and the residue was separated by chromatography on a silica gel column (*n*-hexane : ethyl acetate = 1 : 1) to give the indazole **8** (17 mg, 30 %). ^{1}H NMR (300 MHz, $CDCl_3$): δ 8.80 (d, *J* = 2.0 Hz, 1 H), 8.40 (s, 1 H), 8.32 (dd, *J* = 9.2, 2.1 Hz, 1 H), 7.68 (m, 3 H), 7.30 (t, *J* = 6.1 Hz, 2 H); MS (ESI): *m/z* for $C_{13}H_8FN_3O_2$, found 258 [M+H$^+$].

Preparation of 5-amino-1-(4-fluorophenyl)-1H-indazole (9)

To a solution of compound **8** (17 mg, 0.066 mmol) in dichloromethane (5 mL), 10 % palladium on carbon (10 mg) was added at room temperature, and the mixture was shaken under a hydrogen atmosphere (60~70 psi) at the same temperature for 5 h. The reaction mixture was filtered through Celite and the filtrate was evaporated in vacuo to afford the indazole **9** (14 mg, 93 %). ^{1}H NMR (300 MHz, $CDCl_3$): δ 8.03 (s, 1 H), 7.74 (dd, *J* = 8.1, 5.2 Hz, 2 H), 7.55 (d, *J* = 8.9 Hz, 1 H), 7.38 (t, *J* = 8.7 Hz, 2 H), 6.88 (t, *J* = 8.2 Hz, 2 H), 5.00 (s, 1 H); MS (ESI): *m/z* for $C_{13}H_{10}FN_3$, found 228 [M+H$^+$].

Preparation of 1-(4-fluorophenyl)-5-(4-methoxybenzenesulfonamido)-1H-indazole (4, TRT-0173)

4-methoxybenzenesulfonyl chloride (8 mg, 0.04 mmol) was added to a solution of compound **9** (7 mg, 0.03

mmol) and pyridine (4 µL, 0.05 mmol) in dichloromethane (2 mL) at room temperature, and the mixture was stirred at the same temperature for 15 h. The reaction mixture was partitioned between dichloromethane and water, and the organic layer was dried over magnesium sulfate. The solvent was evaporated in vacuo and the residue was chromatographed on a silica gel column (*n*-hexane : ethyl acetate = 4 : 1) to give the final product **4** (8 mg, 70 %). ^{1}H NMR (500 MHz, $CDCl_3$): δ 8.13 (s, 1 H), 7.69-7.64 (m, 4 H), 7.61 (d, *J* = 9.0 Hz, 1 H), 7.50 (d, *J* = 1.8 Hz, 1 H), 7.25 (t, *J* = 8.6 Hz, 2 H), 7.17 (dd, *J* = 9.0, 2.0 Hz, 1 H), 6.91 (dd, *J* = 7.2, 1.7 Hz, 2 H), 6.55 (s, 1 H), 3.85 (s, 1 H); MS (ESI): *m/z* for $C_{20}H_{16}FN_3O_3S$, found 398 [M+H$^+$].

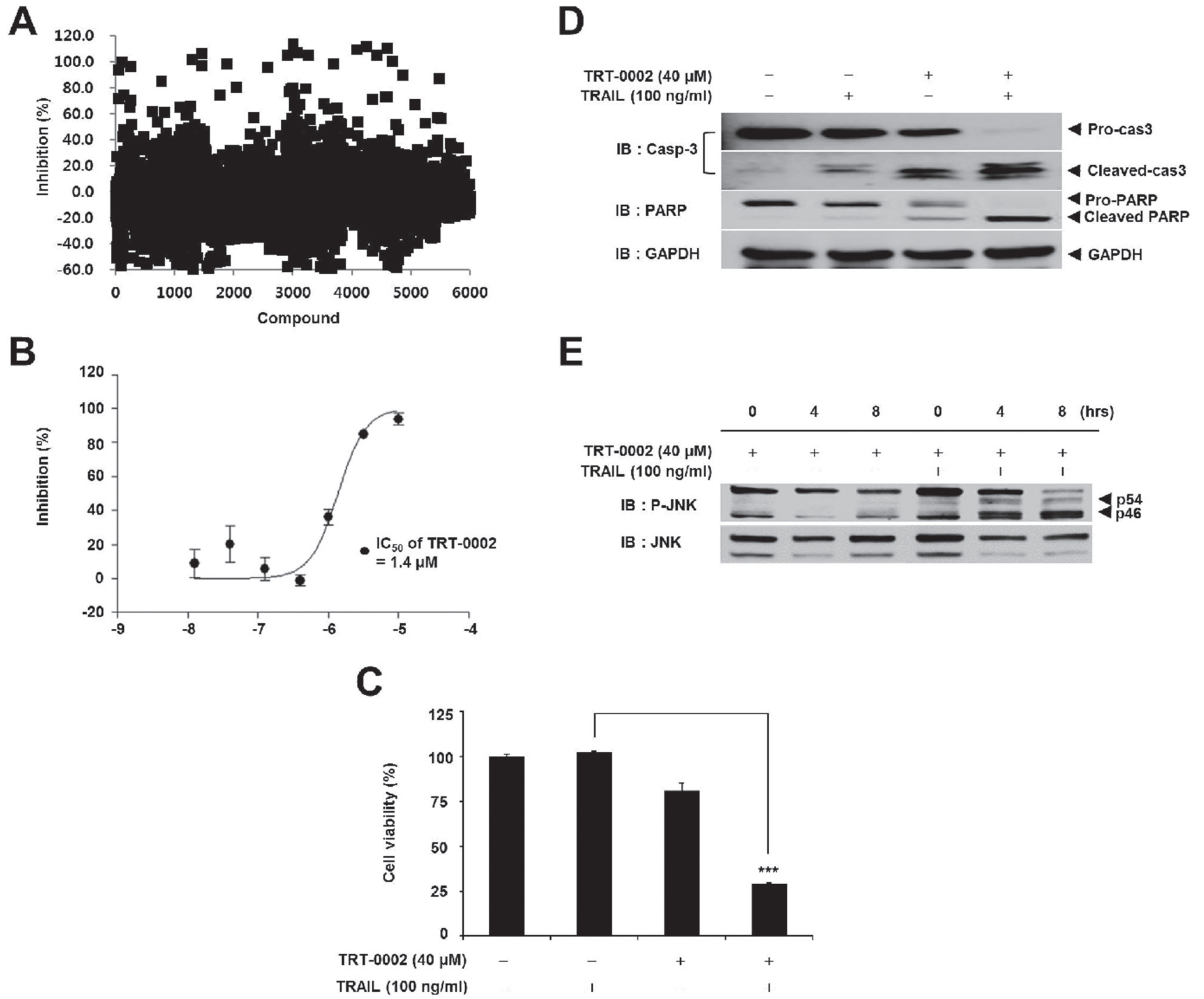

Supplementary Figure 1: The hit chemical compound, TRT-0002, enhances TRAIL-induced apoptosis by inhibiting MKK7-TIPRL interaction. (**A**) ELISA analysis results of 6,000 compounds. (**B**) Inhibition of the MKK7-TIPRL interaction by TRT-0002 using an ELISA system. (**C**) MTT assay for the viability of Huh7 cells treated with TRAIL, TRT-0002, or a combination of TRAIL and TRT-0002 for 48 h. Differences in the viabilities of treated cells were assessed using Student's t-test ($^{***}p < 0.001$). (**D**) Huh7 cells were treated with TRT-0002 (40 µM) and TRAIL (100 ng/ml) for 48 h. The levels of PARP and caspase-3 expression were examined by Western blotting. GAPDH was used as a loading control. (**E**) Huh7 cells were treated with 40 µM TRT-0002 in the presence or absence of 100 ng/ml TRAIL for the indicated time periods. The phosphorylation of JNK was examined by Western blotting.

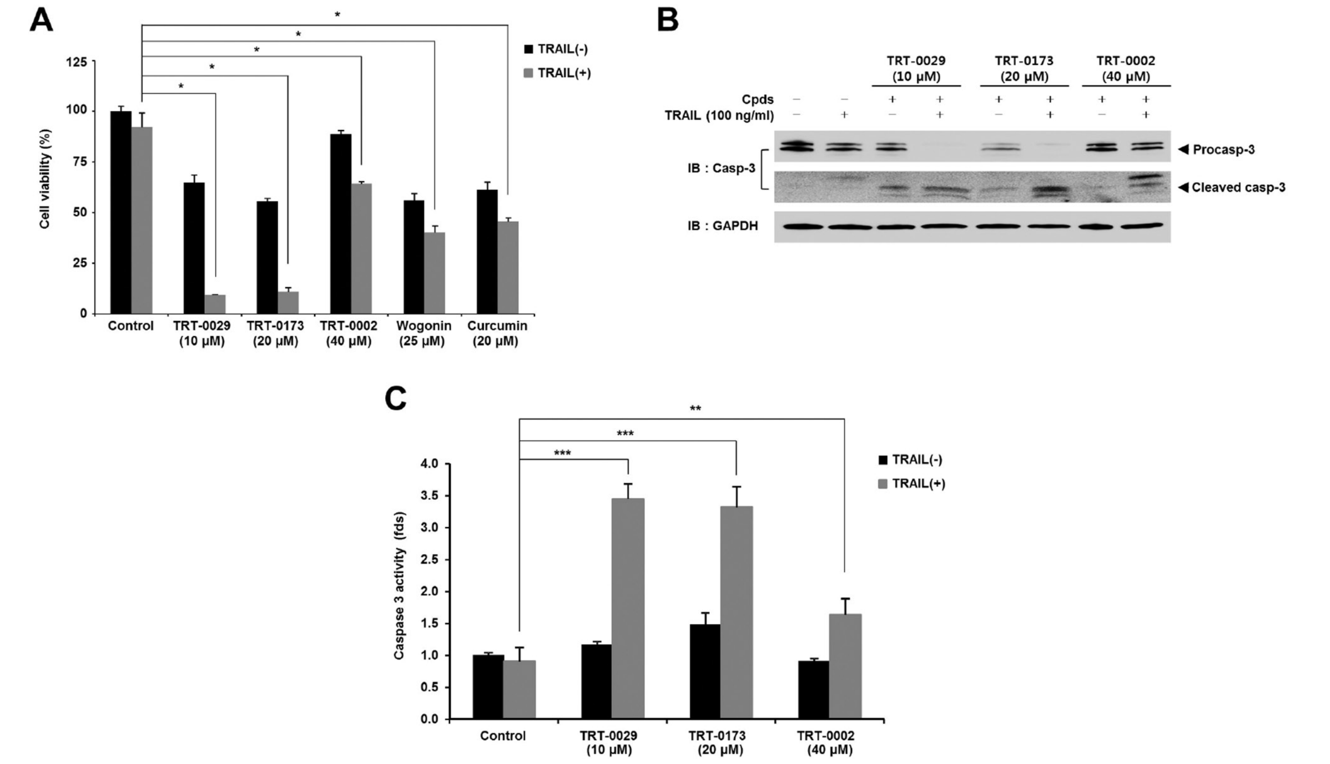

Supplementary Figure 2: Lead compounds (TRT-0029 and TRT-0173) induce higher TRAIL mediated-apoptosis than hit chemical compound (TRT-0002) does. (**A**) Huh7 cells were treated with lead compounds (TRT-0029 and TRT-0173), hit chemical compound (TRT-0002), or reported TRAIL sensitizer (wogonin or curcumin) in the absence or presence of TRAIL (100 ng/ml) for 24 h, and then a MTT assay was performed. The differences in cell viabilities were assessed using Student's t-test ($^{*}p < 0.05$). (**B**) Huh7 cells were treated with the indicated compounds and/or TRAIL for 24 h. The caspase-3 levels were examined by Western blotting. GAPDH was used as a loading control. (**C**) Differences in caspase-3 activity of Huh7 cells treated with the indicated compounds and/or TRAIL for 24 h were assessed using Student's t-test ($^{**}p < 0.01$, $^{***}p < 0.001$).

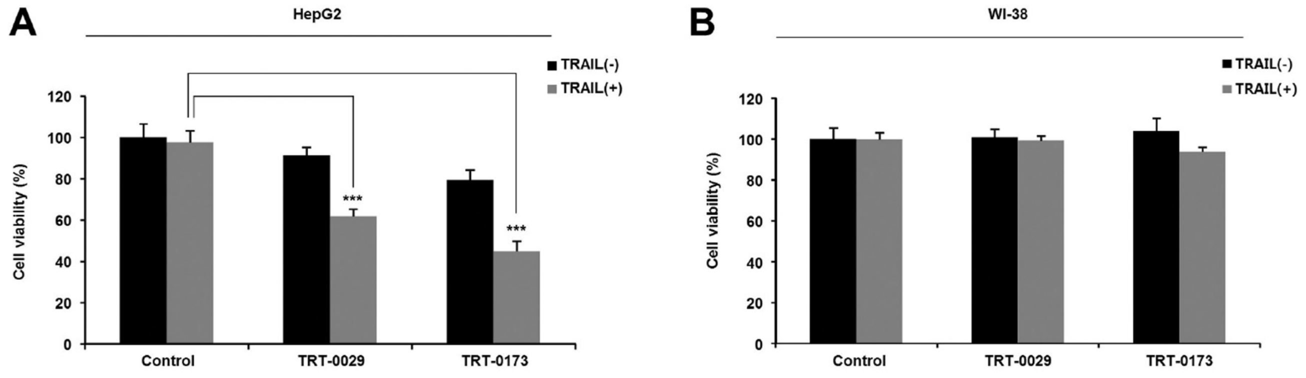

Supplementary Figure 3: Lead compounds enhance TRAIL-induced cell death in HepG2 cells, but not in normal cells. (**A**) MTT assay of HepG2 cells following treatment with lead compounds, TRAIL, or a combination of lead compounds (10 µM for TRT-0029 and 20 µM for TRT-0173) and TRAIL (100 ng/ml) for 24 h. Differences in cell viabilities were assessed using Student's t-test ($^{***}p < 0.001$). (**B**) WI-38 cells were treated with lead compounds and/or TRAIL for 24 h. The MTT assay was performed to measure cell viability.

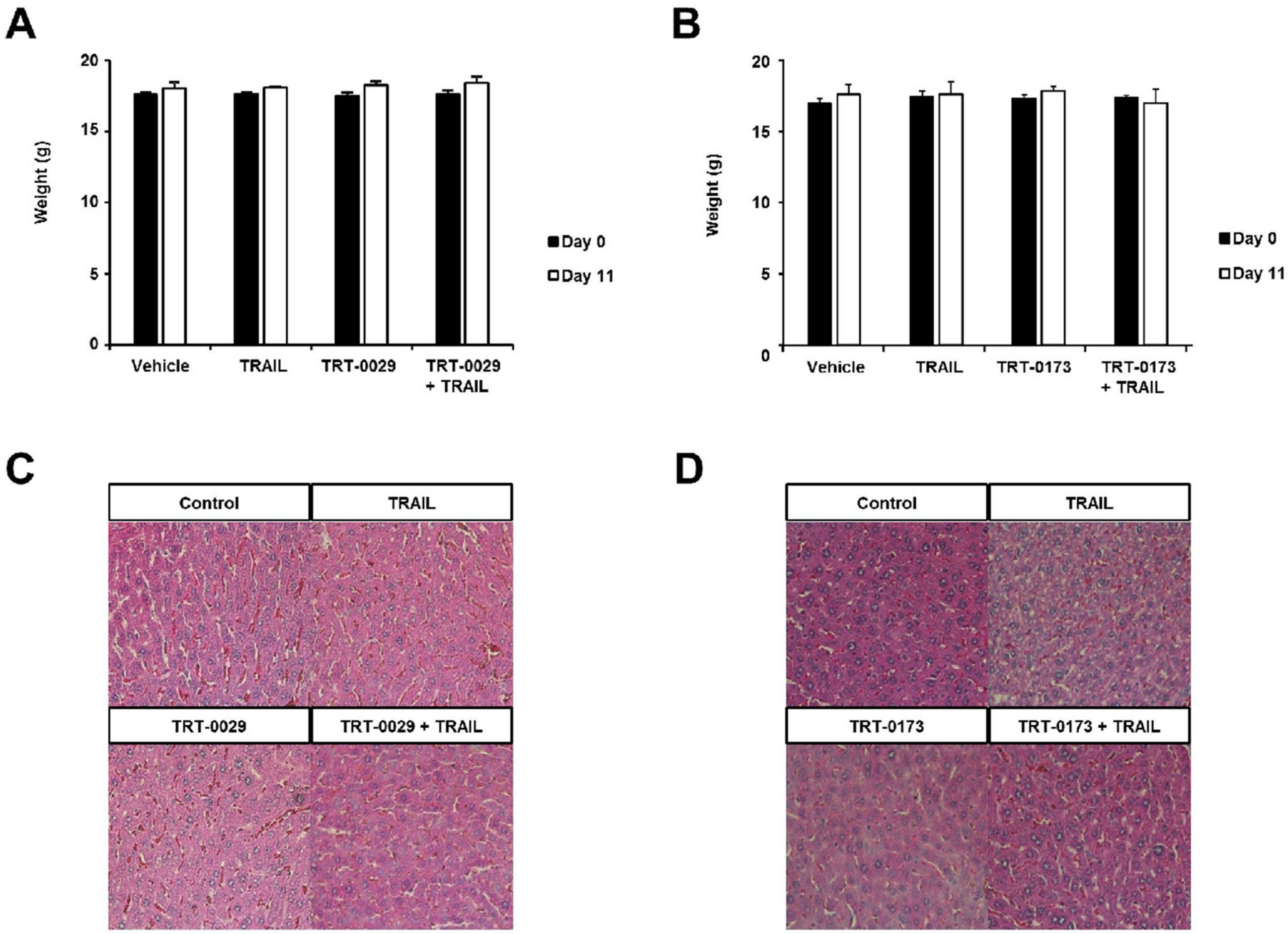

Supplementary Figure 4: Treatments with lead compounds and TRAIL do not show general cytotoxicity in the Huh7 tumor xenograft model. (**A**) The body weights of mice at 0 and 11 days. (**B**) Representative image of H&E stained liver tissues after 11 days. Pictures were taken at a magnification of × 400.

Supplementary Table 1: Average Z'factor of over 6,000 chemical compounds in an in-house chemical library

Plate number	Z' factor[a]	Plate number	Z' factor
1	0.52	11	0.57
2	0.79	12	0.58
3	0.66	13	0.51
4	0.67	14	0.53
5	0.47	15	0.60
6	0.59	16	0.70
7	0.50	17	0.76
8	0.50	18	0.50
9	0.65	19	0.71
10	0.66	20	0.63

[a]Z'factor' = 1-((3 × SD of sample + 3x SD of control)/(mean of sample – mean of control))
SD: standard deviation

Supplementary Table 2: Screening of hit compounds using ELISA and MTT assay

Compounds	ELISA IC_{50} (µM)	Cell viability assay (%)	
		Compound only	Compound + TRAIL
1	2.7	88	87
2	5.9	89	42
3	1.7	42	22
4	0.089	86	91
5	1.4	81	79
6	2.0	93	99
7	8.9	89	88
8	2.8	69	84
9	1.0	90	96
10	3.5	111	113
11	3.6	86	97
12	0.37	30	24
13	5.1	87	89
14	19.8	N.D	N.D
15	0.77	91	95
16	1.0	80	51
17	0.89	96	95
18	12.7	89	86
19	0.036	6	6
20	3.5	102	113
21	0.15	84	80
22	4.7	59	10
23	7.5	87	82
24	17.2	N.D	N.D
25	0.34	93	60
26	2.7	97	91
27	1.3	40	40

IC_{50}: half maximal inhibitory concentration
N.D: Not Determined

Supplementary Table 3: Selection of the lead compounds (TRT-0029 and TRT-0173) from 280 analogs of TRT-0002

Compound	Cell viability assay				Apoptotic protein detection[a]	Caspase 3 activity	JNK phosphorylation[b]	Inhibition assay of MKK7-TIPRL[c]
	Woking concentration (μM)	Treatment time (hrs)	Compound only (%)	Compound + TRAIL (%)		Compound + TRAIL/Compound (ratio)		
TRT-0002	40	48	81	29	+++	1.80	++	++
TRT-0004	40	24	84	34	+++	2.58	ND	
TRT-0006	40	24	62	23	+++	1.01		
**TRT-0029**	**10**	**24**	**69**	**11**	**+++**	**2.20**	+++	+++
TRT-0030	20	24	51	23	ND			
TRT-0049	40	24	61	25	+++	3.81	ND	
TRT-0051	40	24	54	28	+++	1.67		
TRT-0060	40	24	50	36	ND			
TRT-0061	40	24	85	69	+++	2.94	ND	
TRT-0062	40	24	86	64	+++	1.69		
TRT-0065	40	24	88	72	ND			
TRT-0083	40	24	52	36	+++	1.67		
TRT-0086	40	24	59	43	+++	5.71	ND	
TRT-0088	40	24	50	34	+++	4.77	ND	
TRT-0105	10	24	88	48	+	5.01	ND	
TRT-0106	40	24	42	24	+++	5.48	ND	
**TRT-0173**	**10**	**24**	**53**	**7**	**+++**	**2.10**	+++	+++
TRT-0208	20	24	92	6	+++	1.21		
TRT-0211	20	24	87	4	+++	1.47		

a : Expression levels of cleaved caspase-3 and PARP on western blotting (Compound + TRAIL/TRAIL)
b : Phosphorylation levels of JNK on western blotting (Compound + TRAIL/Compound)
c : Inhibition activities of MKK7-TIPRL on GST pull-down assay (Compound + TRAIL/TRAIL)
